# Atorvastatin enhances LDL receptor-mediated LDL-C uptake and modulates PCSK9 protein expression in pancreatic β-cells

**DOI:** 10.1080/19382014.2025.2479906

**Published:** 2025-03-16

**Authors:** Zhiyan Zhang, Huadong Zheng, Lusha Zhang, Peihong Su, Xiaochang Chen, Aoqi Xiang, Juan Yang, Hua Guan, Jianglin Fan, Qi Yu

**Affiliations:** a Engineering Research Center of Brain Health Industry of Chinese Medicine, Key Laboratory of Pharmacodynamics and Material Basis of Chinese Medicine of Shaanxi Administration of Traditional Chinese Medicine, Department of Pharmacology, Shaanxi University of Chinese Medicine, Xianyang, China; b Shaanxi Key Laboratory of Ischemic Cardiovascular Diseases and Institute of Basic and Translational Medicine, Xi’an Medical University, Xi’an, China; c Department of Gerontology, The Second Affiliated Hospital of Xi’an Jiaotong University, Xi’an, China; d School of Biotechnology and Health Sciences, Wuyi University, Jiangmen, China

**Keywords:** LDL receptor, low-density lipoprotein cholesterol, pancreatic β-cell, proprotein convertase subtilisin/kexin type 9, statin, type 2 diabetes

## Abstract

Statins are widely used to treat hyperlipidemia and atherosclerotic cardiovascular diseases (ACVD) by significantly lowering low-density lipoprotein cholesterol (LDL-C) levels. However, their use has been associated with an increased risk of type 2 diabetes (T2D), a paradox given their lipid-lowering benefits. This study investigates the role of LDL receptors (LDLR) and proprotein convertase subtilisin/kexin type 9 (PCSK9) in the diabetogenic effects of atorvastatin on pancreatic β-cells. Using the MIN6 pancreatic β-cell line, we assessed the impact of atorvastatin on LDL-C uptake, PCSK9 expression, glucose-stimulated insulin release (GSIR), and cell proliferation. Cellular cholesterol assays, EdU labeling, Dil-LDL uptake, western blot analysis, reverse transcription-quantitative polymerase chain reaction (RT-qPCR), and ELISA, were employed to measure relevant biomarkers and cellular responses. Male C57BL/6j mice were treated with atorvastatin to validate in vitro findings. Atorvastatin enhances LDL-C uptake by upregulating LDLR on the cell surface, without causing excess cholesterol accumulation. Additionally, atorvastatin suppresses PCSK9 expression, which is crucial for LDLR degradation. Interestingly, atorvastatin, combined with exogenous LDL-C, impairs glucose-stimulated insulin release (GSIR) but promotes cell proliferation, highlighting a potential mechanism for statin-associated diabetes. Oral administration of atorvastatin in mice reduced plasma PCSK9 and insulin levels, supporting the in vitro findings. These results indicate that while atorvastatin effectively lowers circulating cholesterol, it may adversely affect pancreatic β-cell function by modulating LDLR and LDL-C uptake, thereby increasing the risk of T2D. This study highlights the importance of further research to develop strategies mitigating the diabetogenic effects of statins while maintaining their cardiovascular benefits.

## Introduction

Statins are the first-line agents for treating hyperlipidemia and atherosclerotic cardiovascular diseases (ACVD), and they can lower LDL-C levels by as much as 60%.^1^ Statins reduce the relative risk of major coronary events by approximately 30%, and numerous ACVD patients treated with statins receive absolute benefits worldwide.^2^ In addition to their lipid-lowering potency, statins have other beneficial effects on ACVD, such as improved endothelial function, reduced plaque vulnerability, inhibited myocyte infiltration, and reduced inflammation.^3^ Despite statins being highly effective and safe for most people, statins can have adverse effects on some patients, causing muscle pain and damage, liver damage, and an increased risk of type 2 diabetes (T2D).^4^ Notably, statin-associated T2D has received extensive attention because people with ACVD are often accompanied by T2D.^5^ Hyperlipidemia is a risky factor for T2D patients, and lipid-lowering therapy with statins is supposed to decrease the risk of T2D.^6^ Paradoxically, statin usage is associated with a higher risk of T2D and an increased risk of hyperglycemic complications.^7^

In recent years, numerous clinical studies have suggested a causal relationship between statin use and new-onset diabetes (NOD), which has greatly impacted the clinical use of statins.^7,8^ To solve this puzzle, we have speculated that low-density lipoprotein receptor (LDLR) may play a role in the pathological process of statin-associated T2D.^9,10^ Briefly, statins may enhance LDL-C intake by pancreatic islets via upregulation of LDLR; subsequently, exogenous cholesterol may impair the function of pancreatic islets, resulting in an increased risk of T2D. However, LDLR expression alone does not fully explain the observed effects of statins on β-cell function. PCSK9, a key regulator of LDLR degradation, is highly expressed in pancreatic β-cells and may influence cholesterol homeostasis and insulin secretion.^11^ Given that statins suppress PCSK9 expression in the liver, it is critical to determine whether similar regulatory mechanisms occur in pancreatic β-cells and whether PCSK9 plays a role in the diabetogenic effects of statins. More importantly, because the effects of statins on the function of pancreatic islets and their regulation of LDLR are not fully understood, there is no efficient method for preventing the onset of statin-associated T2D. To address these questions, we used atorvastatin to incubate a pancreatic β-cell line (MIN6), exploring the underlying mechanism of atorvastatin-induced LDLR regulation and how LDLR-mediated LDL-C uptake affects the function of MIN6 cells.

## Materials and methods

### Animals

The study was conducted following the Basic & Clinical Pharmacology & Toxicology policy for experimental and clinical studies.^12^ Animal experiments in this study were conducted by the Guidelines for Animal Experimentation of Xi’an Medical University (Xi’an, China). The experimental protocols were approved by the Laboratory Animal Administration Committee of Xi’an Medical University (Institutional Animal Care and Use Committee; permit no. XYJZS-2024032105). Male C57BL/6j mice (12 weeks old; ~30 g) were purchased from the laboratory animal center at Xi’an Jiaotong University (Xi’an, China). A total of 12 C57BL/6j mice were randomly divided into two groups and received a normal chow diet. Referring to a previous study,^13^ one group was treated with atorvastatin via intragastric administration (10 mg/kg per day; Sigma-Aldrich; Merck KGaA, Darmstadt, Germany) for 4 weeks; the other group received the same dose of double distilled water (*n* = 6; respectively). All mice were individually housed in plastic cages (30 × 20 × 13 cm) throughout the study and maintained on a 12/12 h light/dark cycle (lights off at noon) at a constant temperature of 22°C with 10–15 h cycles of fresh air and relative humidity (60 ± 10%). Food and water were available ad libitum.

### Cell culture and cellular cholesterol assay

The experiments were designed to investigate the effect of atorvastatin on LDL receptor-mediated LDL-C uptake and its modulation of PCSK9 expression in pancreatic β-cells. LDLR upregulation was hypothesized to enhance cholesterol intake, potentially impacting glucose-stimulated insulin release (GSIR), and contributing to the diabetogenic effects observed with statin use. Mouse pancreatic β-cell line (MIN6 cells) was purchased from BioTNT (BioTNT, Shanghai, China). Cells were cultured in DMEM containing 15% FBS, 25 mmol/L glucose, 50 µmol/L 2-mercaptoethanol, 100 U/mL penicillin, and 100 µg/mL streptomycin. After the designated treatments, cellular assays such as cholesterol quantification, EdU proliferation assays, and Dil-LDL uptake assays were performed. Western blotting and immunofluorescence techniques were employed to measure LDL receptor (LDLR) and proprotein convertase subtilisin/kexin type 9 (PCSK9) protein expression. The cells were maintained at 37°C in a humidified atmosphere of 95% air and 5% CO2. MIN6 cells (10^4^) were seeded in 12-well plates and treated with atorvastatin hemicalcium salt sesquihydrate (0, 1, 10, and 100 nM; Sigma‑Aldrich, Merck KGaA, Germany) for 24 h with or without LDL-C (10 ug/mL; Yiyuan Biotechnologies, Guangzhou, China) (*n* = 3; respectively), referring to a previous study.^14^

The medium was discarded and washed twice with PBS, and cholesterol measurements were performed immediately after treatment. The kits (Solarbio, Shanghai, China) were used for monitoring cellular total cholesterol (TC), free cholesterol (FC), and cholesterol ester (CE) (*n* = 3; each simple was performed with 3 technical replicates, respectively). MIN6 cells were washed with cold PBS to remove the residual medium. Cells were then lysed using a cell lysis buffer and incubated on ice for 30 minutes to ensure complete lysis. The lysate was collected, transferred to a microcentrifuge tube, and centrifuged at 10,000 × g for 10 minutes at 4°C to remove cell debris. The supernatant was carefully collected and mixed with the cholesterol assay reagent as per the kit instructions. The reaction mixture was incubated at 37°C for 30 minutes, and the absorbance was measured at 500 nm using a microplate reader. Cholesterol concentrations were determined by comparing the absorbance readings to a cholesterol standard curve. For quantitative analysis, each condition was assessed with three biological replicates, and three technical replicates were performed for each sample to ensure accuracy and minimize variation.

### EdU labeling assay

MIN6 cells (1–4 × 10^4^/well) were seeded in 96-well plates and treated with atorvastatin (0, 1, 10, and 100 nM) for 24 h with or without LDL-C (10 ug/mL). EdU was added to the culture medium at a concentration of 10 µM for 2 hours. Cell proliferation was assessed using the Cell-Light EdU DNA cell proliferation kit (RiboBio, Guangzhou, China) following the manufacturer’s instructions. The staining was visualized using Nikon TE2000 Inverted Fluorescence Microscope (Nikon Corporation, Tokyo, Japan), and the red fluorescence was deemed as EdU staining-positive cells.

### Dil-LDL uptake assay

MIN6 cells were cultured and inoculated in confocal dishes, then incubated with atorvastatin (0 and 10 nM) for 24 hours. Following this, the cells were switched to serum-free media and incubated with 10 μg/ml Dil-LDL (Yiyuan Biotechnologies, Guangzhou, China) for 24 hours at 37°C in the dark. After incubation, the cells were washed with PBS, fixed in 4% paraformaldehyde, and their nuclei stained with DAPI. Finally, the cells were examined by using Nikon TE2000 Inverted Fluorescence Microscope (Nikon Corporation, Tokyo, Japan), and the red fluorescence was deemed as Dil-LDL staining-positive cells.

### Protein extraction and western blot analysis

Total protein was extracted from MIN6 cells as described.^15^ Primary antibodies used were: LDLR (1:500; Abcam, Cambridge, UK), PCSK9 (1:1000; Abcam), inducible degrader of LDL receptor (IDOL) (1:2000; Sigma-Aldrich; Merck KGaA), and GAPDH (1:2000; Santa Cruz Biotechnology, Inc.). The membranes were incubated with these primary antibodies overnight at 4°C. Subsequently, they were incubated with horseradish peroxidase-conjugated secondary antibodies (1:2500; Beyotime Institute of Biotechnology, Haimen, China) for 3 hours at room temperature. Western blot analysis was performed as previously described,^16^ and relative protein expression was quantified using ImageJ (bundled with Java 1.8.0_172; NIH). Repeat experiments can be found in Supplementary Material.

### RNA extraction and reverse transcription‑quantitative polymerase chain reaction

The total RNA of MIN6 cells was extracted using RNAiso Plus (Takara Bio, Inc., Otsu, Japan) (*n* = 3; respectively). RT‑qPCR was performed and quantified using the 2^‑ΔΔCq^ method as previously described (*n* = 3; respectively).^16^ The sequences of the primers are listed as follows: LDLR, 5'‑TGACCTTCATCCCAGAGCCTTC‑3' and 5'‑GGCATGAGCGGGTATCCATC‑3;' proprotein convertase subtilisin/kexin type 9 (PCSK9), 5'‑TATCCCAGCATGGCACCA GA‑3' and 5'‑ATGGTGACCCTGCCCTCA A‑3;' E3 ubiquitin‑protein ligase MYLIP (IDOL), 5'‑AGGAGATCAACTCCACCTTCT G‑3' and 5'‑ATCTGCAGACCGGACAGG‑3; GAPDH, 5'‑ACTGAGGACCAGGTTGTC‑3' and 5'‑TGCTGTAGCCGTATTCATTG‑3.'

### ELISA

Plasma PCSK9 and insulin levels were measured using commercial ELISA kits (cat. no. MPC900; Mouse Quantikine ELISA for PCSK9; R&D Systems, Inc., Minneapolis, MN, USA; cat. no. YK060; Insulin ELISA; Yanaihara Institute Inc., Fujinomiya, Japan) during the 4th week of intervention, as previously described.^13^ For quantitative analysis, each condition was assessed with three biological replicates, and technical replicates were performed in triplicate for each sample to ensure accuracy and minimize variation.”

### Immunofluorescence

After 48 hours of incubation, MIN6 cells were fixed with 4% formaldehyde for 1 hour at room temperature, followed by blocking with 5% bovine serum albumin (Beyotime Institute of Biotechnology) in PBS for 1 hour at room temperature. To prevent the detection of internalized LDLR in intracellular lysosomes, cells were not permeabilized with Triton X-100 before incubation. The islets were then incubated overnight at 4°C with primary antibodies (1:500; Abcam, Cambridge, UK) targeting LDLR. Subsequently, Alexa Fluor 488 secondary antibodies (1:2000; Thermo Fisher Scientific, Inc.) were applied for 3 hours at room temperature. Images were captured using a laser scanning confocal microscope (FV1200, Olympus, Japan) and analyzed with ImageJ software, as described previously.^13^

### Statistical analysis

Before statistical analysis, normality was assessed using the Shapiro-Wilk test, and homogeneity of variance was evaluated with Brown-forsythe test (Supplementary Material 2). Results confirmed the data met parametric assumptions (supplementary material). All data are expressed as the mean ± SE. Two groups of comparisons were used by Student’s t-test. Multiple groups of comparisons were performed by using one-way ANOVA with the Bonferroni test. Two-way ANOVA with Bonferroni posttest was used to evaluate the effects of combined atorvastatin and LDL-C on GSIR. *p* < 0.05 was considered statistically significant. The statistical calculations were performed by using SPSS 19.0 software (IBM Corp., Armonk, NY, USA). Group allocations were made by an independent researcher. Both the experimenters and outcome assessors were blinded to the group assignments. Data analysis was performed by a statistician who was also blinded to group allocations.

## Results

### Atorvastatin enhances LDL-C uptake without causing excess cholesterol accumulation in MIN6 cells

**Figure 1.**
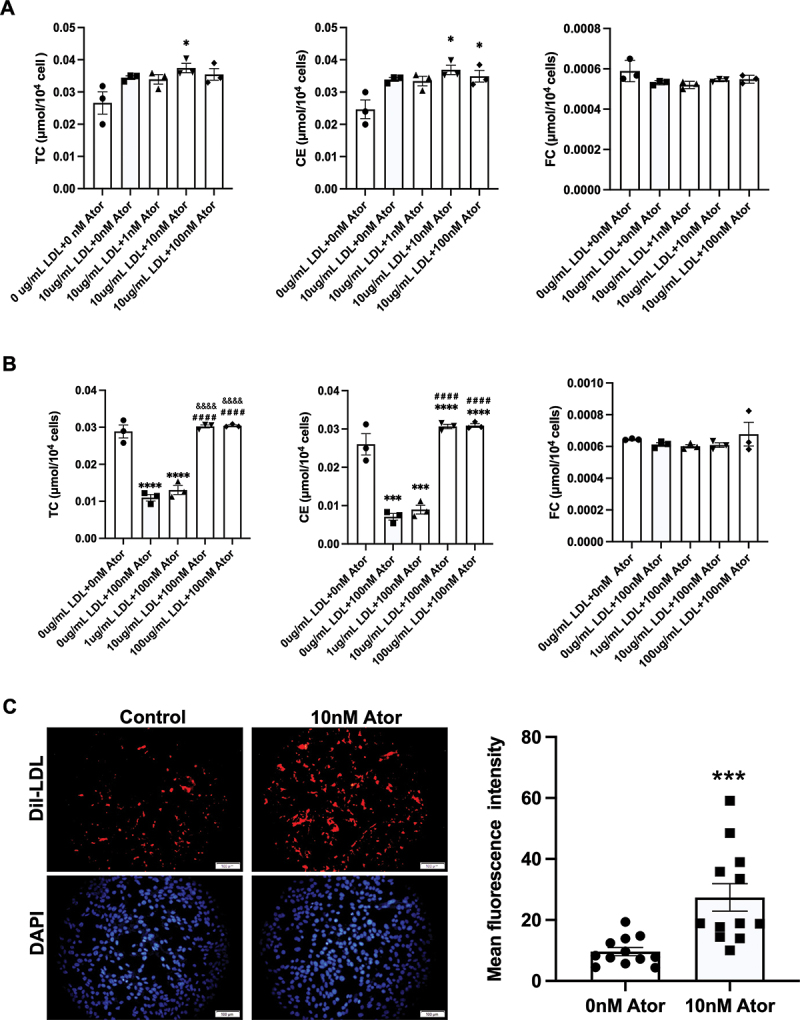
Effect of atorvastatin on cholesterol synthesis and uptake in pancreatic β-cells. (A) Total cholesterol (TC), cholesterol ester (CE), and free cholesterol (FC) were measured in MIN6 cells after incubation of combined atorvastatin (0, 1, 10, and 100 nM) with LDL-C (10 ug/mL) for 24 h. (B) TC, CE, and FC were measured in MIN6 cells after incubation of combined atorvastatin (100 nM) with LDL-C (0, 1, 10, and 10 ug/mL) for 24 h. (C) LDL uptake was assessed by fluorescence microscope after incubation of combined atorvastatin (10 nM) with Dil-LDL-C (10 ug/mL) for 24 h (n = 12). The effects of combined atorvastatin and LDL-C were analyzed by using two-way ANOVA with the Bonferroni test, and Student’s t-test was used to evaluate the comparisons of LDL uptake. The data are expressed as the mean ± S.E.M. *p < 0.05, ***p < 0.001 and ****p < 0.0001 versus the control group (0 ug/mL LDL +0 nM Ator). ^####^*p* < 0.0001 versus the group (0 ug/mL LDL +100 nM Ator). ^&&&&^*p* < 0.0001 versus the group (1 ug/mL LDL +100 nM Ator).

To assess the effect of atorvastatin on cholesterol synthesis and uptake in pancreatic β-cells, we measured TC, CE, and FC after incubating MIN6 cells with various concentrations of atorvastatin and LDL-C. As shown in Figure 1A, with a 10 µg/mL dose of LDL-C (exogenous cholesterol) presenting in DMEM, a 10 nM dose of atorvastatin increased TC and CE levels in MIN6 cells compared with the control group, and a 100 nM dose of atorvastatin only increased the CE level compared with the control group. However, atorvastatin did not affect the cellular FC levels (Figure 1A). Statins can promote exogenous cholesterol uptake by upregulating LDLR while suppressing endogenous cholesterol synthesis by inhibiting HMG-CoA reductase (HMGCR). This dual effect is dose-dependent and influenced by adding LDL-C. As shown in Figure 1B, without presenting LDL-C or addition of 1 ug/mL LDL-C, a 100 nM dose of atorvastatin dramatically reduced TC and CE levels compared with the control group, showing that atorvastatin indeed suppresses endogenous cholesterol synthesis. However, when LDL-C concentrations increased to 10 µg/mL, TC and CE levels returned to normal compared to the control group, indicating that adding exogenous cholesterol neutralizes atorvastatin-suppressed endogenous cholesterol synthesis. Whereas, adding 100 µg/mL LDL-C, it did not cause excess cholesterol accumulation in MIN6 cells with a 100 nM dose of atorvastatin incubation (Figure 1B). To further verify atorvastatin-promoted exogenous cholesterol uptake, MIN6 cells were incubated with a 10 nM dose of atorvastatin and a 10 µg/mL dose of Dil-LDL. As shown in Figure 1C, atorvastatin dramatically increased Dil-LDL uptake (Figure 1C).

### Differential effects of atorvastatin on MIN6 cell proliferation in the presence of LDL-C

**Figure 2.**
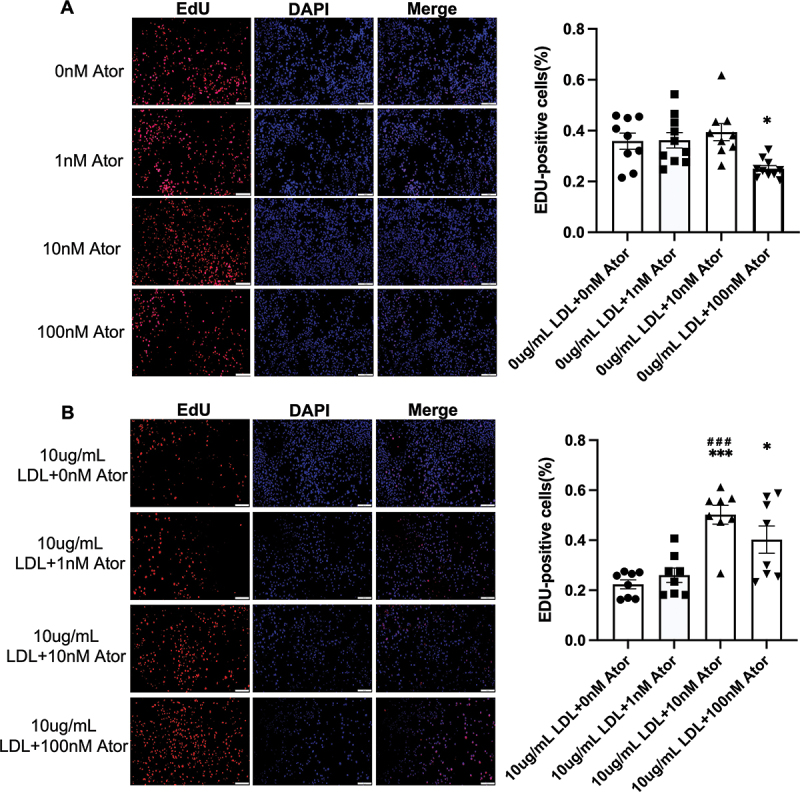
Atorvastatin affects the proliferation of MIN6 cells. (A) EdU staining assesses the effect of atorvastatin (0, 1, 10, and 100 nM) on MIN6 cells proliferation (left) and quantification of EdU staining-positive cells (right) (n = 8). (B) EdU staining assesses the effect of combined atorvastatin (0, 1, 10, and 100 nM) with LDL-C (10 ug/mL) on MIN6 cells proliferation (left) and quantification of EdU staining-positive cells (right) (n = 8). The effects of combined atorvastatin and LDL-C were analyzed by using two-way ANOVA with the Bonferroni test. The data are expressed as the mean ± S.E.M. *p < 0.05 versus the control groups (0 ug/mL LDL +0 nM Ator or 10 ug/mL LDL +0 nM Ator); ^###^*p* < 0.001 versus the group (0 ug/mL LDL +1 nM Ator).

Given that cell proliferation is a prerequisite for pancreatic islet function, we used EdU staining to assess the effect of atorvastatin on the proliferation of MIN6 cells. As shown in Figure 2A, a 100 nM dose of atorvastatin significantly suppressed MIN6 cell proliferation. In contrast, the co-incubation of atorvastatin (10 nM and 100 nM) and a 10 µg/mL dose of LDL- significantly promoted the proliferation of MIN6 cells.

### Atorvastatin impairs the glucose-stimulated insulin release (GSIR) with the addition of LDL-C

**Figure 3.**
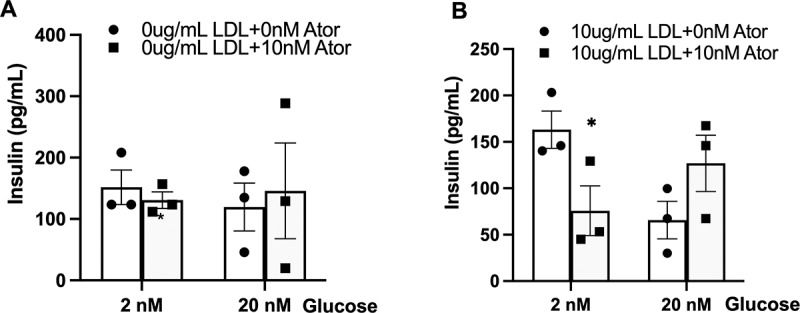
The effect of atorvastatin on the glucose-stimulated insulin release (GSIR) in MIN6 cells. (A) Glucose (2 and 20 nM) stimulates insulin secretion in MIN6 cells after incubation of atorvastatin (10 mm) for 24 h (n = 3). (B) Glucose (2 and 20 nM) stimulates insulin secretion in MIN6 cells after incubation of combined atorvastatin (10 nM) with LDL-C (10 ug/mL) for 24 h (n = 3). Two-way ANOVA with Bonferroni posttest was used to evaluate the effect of atorvastatin on GSIR. The data are expressed as the mean ± S.E.M. *p < 0.05 versus the control groups (10 ug/mL LDL +0 nM Ator).

Next, we performed GSIR experiments to assess whether atorvastatin could disturb insulin secretion. As shown in Figure 3A, a 10 nM dose of atorvastatin did not affect GSIR (2 nM and 20 nM doses of glucose). However, with presenting a 10 µg/mL dose of LDL-C, a 10 nM dose of atorvastatin significantly suppressed GSIR at a 2 nM glucose stimulation (Figure 3B).

### Atorvastatin upregulates LDL receptors in the cytomembrane without affecting total protein levels

**Figure 4.**
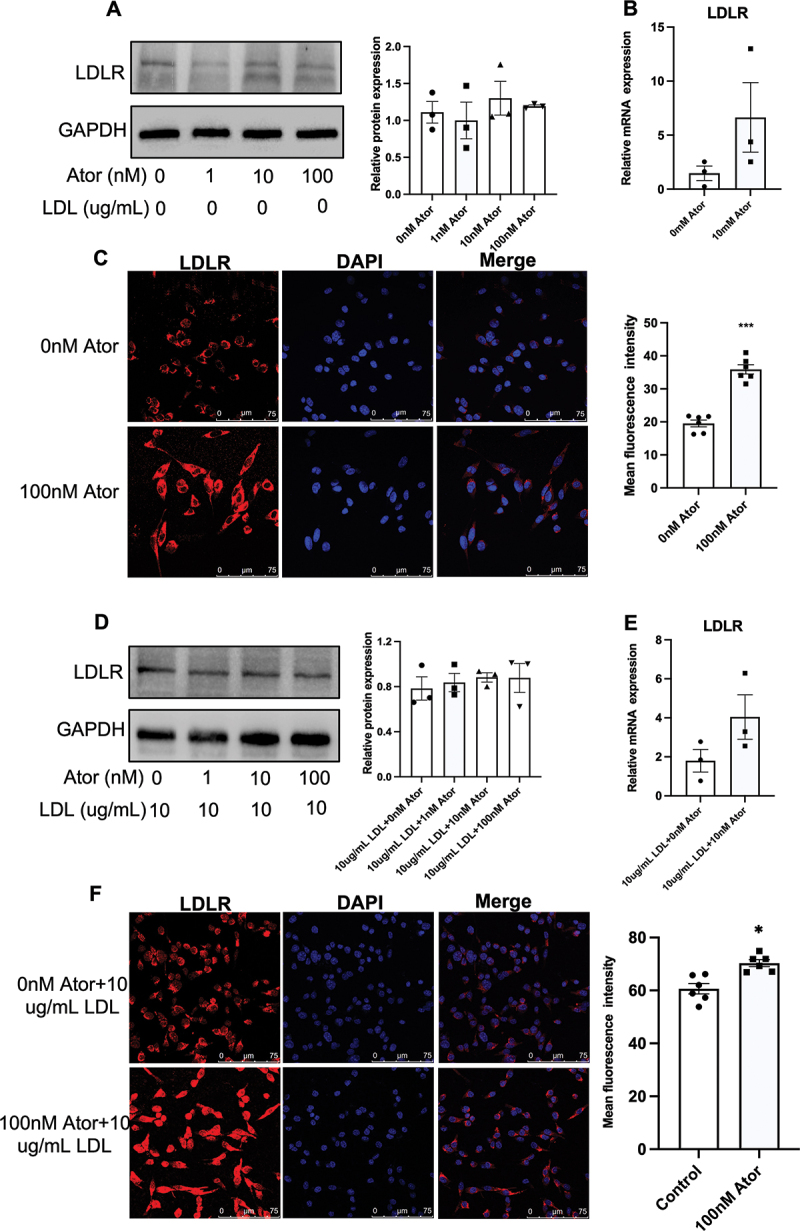
The effect of atorvastatin on the LDLR expression in MIN6 cells. (A) Immunoblot analysis was performed in MIN6 cells after incubation of atorvastatin ((0, 1, 10, and 100 nM) for 24 h (left) and LDLR protein expression was semi-quantified (right; n = 3). (B) mRNA expression of LDLR was confirmed by real-time PCR in MIN6 cells after incubation of atorvastatin (10 nM; n = 3). (C) Immunohistochemistry and confocal microscopy for LDLR protein expression in MIN6 cells cultured with atorvastatin (100 nM) for 24 h and the bar indicates 75 μM (left) and the semi-quantification of LDLR protein expression in MIN6 cells (right; n = 6). (D) Immunoblot analysis was performed in MIN6 cells after incubation of combined atorvastatin ((0, 1, 10, and 100 nM) with LDL-C (10 ug/mL) for 24 h (left) and LDLR protein expression was semi-quantified (right; n = 3). (B) mRNA expression of LDLR was confirmed by real- time PCR in MIN6 cells after incubation of combined atorvastatin (10 nM) with LDL-C (10 ug/m) (n = 3). (C) Immunohistochemistry and confocal microscopy for LDLR protein expression in MIN6 cells cultured with combined atorvastatin (100 nM) with LDL-C (10 ug/mL) for 24 h and the bar indicates 75 μM (left) and the semi-quantification of LDLR protein expression in MIN6 cells (right; n = 6). Multiple groups of comparisons were performed by using one-way ANOVA with the Bonferroni test and the effects of combined atorvastatin and LDL-C were analyzed by using two-way ANOVA with the Bonferroni test and Student’s t-test was used to evaluate the comparisons of two groups. The data are expressed as the mean ± S.E.M. **p < 0.01 and ***p < 0.001 versus the control groups (0 ug/mL LDL +0 nM Ator or 10 ug/mL LDL +0 nM Ator).

Considering that cells can intake exogenous cholesterol via LDLR-mediated internalization of LDL-C, we measured LDLR expression to investigate the mechanism underlying atorvastatin-induced LDL-C uptake. As shown in Figure 4A,B, various doses of atorvastatin did not significantly affect LDLR protein expression, which was further confirmed by Real-Time PCR measurement of mRNA abundance. Next, by using immunofluorescence without membrane permeability reagents, we found that a 100 nM dose of atorvastatin dramatically promoted LDLR protein expression on the cytomembrane (Figure 4C). Similarly, when MIN6 cells were incubated with atorvastatin and a 10 µg/mL dose of LDL-C, three doses of atorvastatin did not affect LDLR protein and mRNA expressions (Figure 4D,E). However, immunofluorescence indicated that atorvastatin could also promote LDLR protein expression on the cell surface (Figure 4F).

### Atorvastatin downregulates PCSK9 expression in MIN6 cells without LDL-C addition

**Figure 5.**
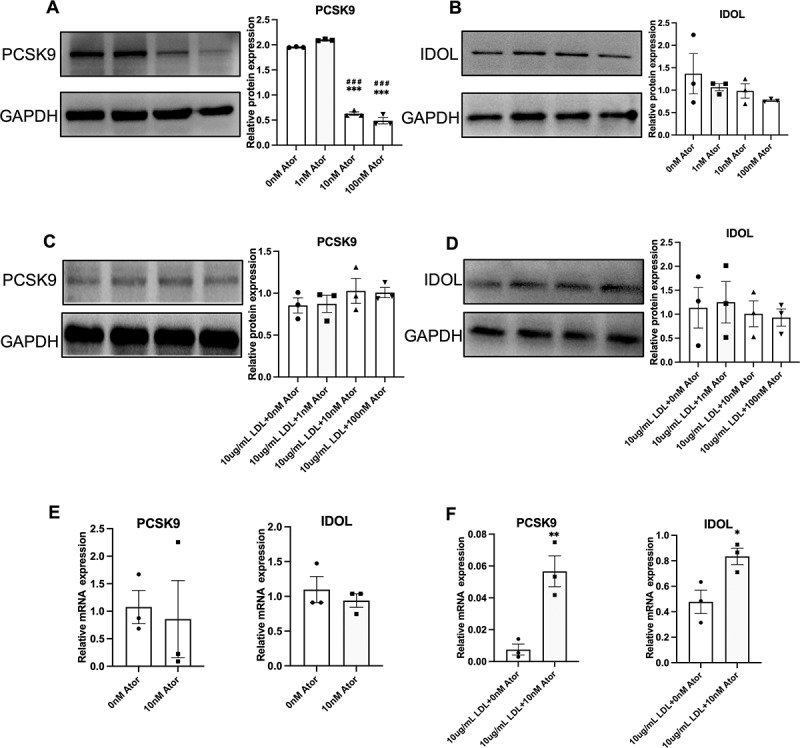
The effect of atorvastatin on the PCSK9 and IDOL expression in MIN6 cells. (A) Immunoblot analysis for PSCK9 protein expression in MIN6 cells after incubation of atorvastatin (0, 1, 10, and 100 nM) for 24 h (left) and the semi-quantification of PCSK9 protein expression (right) (n = 3). (B) Immunoblot analysis for IDOL protein expression in MIN6 cells after incubation of atorvastatin (0, 1, 10, and 100 nM) for 24 h (left) and the semi-quantification of IDOL protein expression (right) (n = 3). (C) Immunoblot analysis for PCSK9 protein expression in MIN6 cells after incubation of combined atorvastatin (0, 1, 10, and 100 nM) with LDL-C (10 ug/mL) for 24 h (left) and the semi-quantification of PCSK9 protein expression (right) (n = 3). (D) Immunoblot analysis for IDOL protein expression in MIN6 cells after incubation of combined atorvastatin (0, 1, 10, and 100 nM) with LDL-C (10 ug/mL) for 24 h (left) and the semi-quantification of IDOL protein expression (right) (n = 3). (E) mRNA expressions of PCSK9 (left) and IDOL (right) were confirmed by real-time PCR in MIN6 cells after incubation of atorvastatin (10 nM) for 24 h n = 3). (F) mRNA expressions of PCSK9 (left) and IDOL (right) were confirmed by real-time PCR in MIN6 cells after incubation of combined atorvastatin (10 nM) with LDL-C (10 ug/m) (n = 3). Multiple groups of comparisons were performed by using one-way ANOVA with the Bonferroni test and the effects of combined atorvastatin and LDL-C were analyzed by using two-way ANOVA with the Bonferroni test and Student’s t-test was used to evaluate the comparisons of two groups. The data are expressed as the mean ± S.E.M^.*^p < 0.05, **p < 0.01 and ***p < 0.001 versus the control groups (0 ug/mL LDL +0 nM Ator or 10 ug/mL LDL +0 nM Ator). ^###^*p* < 0.001 SOR versus the group (0 ug/mL LDL +1 nM Ator).

Protein post-translational modifications determine the LDLR levels on the cell surface, mainly including two pathways as PCSK9 and IDOL-mediated LDLR degradations. Because of that, we studied the PCSK9 and IDOL expression in atorvastatin-treated MIN6 cells. (Figure 5A,B shows, atorvastatin (10 and 100 nM) radically suppressed PCSK9 protein expression, but atorvastatin did not significantly affect IDOL protein expression. Whereas, when MIN6 cells were incubated by atorvastatin with a 10 ug/mL dose of LDL-C, three doses of atorvastatin did not significantly affect either PCSK9 or IDOL protein expression (Figure 5C,D). Of note, a single application of atorvastatin did not affect both PCSK9 and IDOL mRNA expressions in MIN6 cells (Figure 5E), but combined atorvastatin (10 nM) with LDL-C (10 ug/mL) promoted both PCSK9 and IDOL mRNA expression (Figure 5F).

### Oral atorvastatin administration decreases plasma PCSK9 and insulin levels in mice

**Figure 6.**
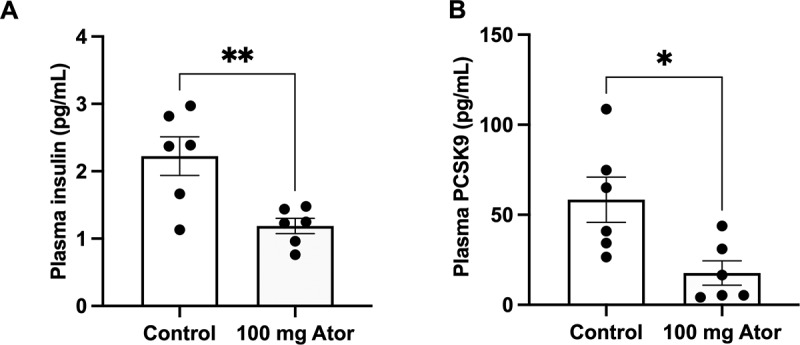
The effect of oral atorvastatin on plasma insulin and PCSK9 levels. (A) Plasma insulin and (B) PCSK9 levels were measured by ELISA kits after oral atorvastatin (100 mg/day) being administrated for 4 weeks (n = 6). Student’s t-test was used to evaluate the comparisons of two groups. The data are expressed as the mean ± S.E.M. *p < 0.05 and **p < 0.01 versus the control groups.

Given that circulating PCSK9 levels are key LDLR regulators for peripheral organs, we measured plasma PCSK9 levels after 4 weeks of oral atorvastatin administration (100 mg/day) in mice. As shown in Figure 6A,B, plasma insulin, and PCSK9 levels were reduced following 4 weeks of atorvastatin administration.

## Discussion

Over the past 20 years, numerous studies have indicated that statin use is associated with T2D, though the underlying mechanism remains unclear.^17^ Previous studies focused on the effects of statins on pancreatic β-cell function, emphasizing insulin pathways, mitochondrial pathways, and oxidative stress pathways.^18^ However, they overlooked that statins are inhibitors of hydroxymethylglutaryl coenzyme A reductase, primarily targeting cholesterol synthesis and influencing cholesterol metabolism. Statins may disturb pancreatic islet function by affecting cholesterol metabolism in β-cells. Given that, our study explores the interplay between statin therapy, specifically atorvastatin, and pancreatic β-cell function under hyperlipidemic conditions. As shown in our findings, atorvastatin suppresses endogenous cholesterol synthesis in MIN6 cells, but the addition of LDL-C neutralizes this effect. Interestingly, despite atorvastatin promoting LDL-C uptake, cellular cholesterol levels do not become excessively high. Given there are some negative feedback mechanisms by the SREBP-2 and HMGCR pathways,^19^ pancreatic β-cells may regulate their intracellular cholesterol levels thereby lowering cholesterol synthesis. Despite that, a certain degree of cholesterol accumulation may impair β-cell function and insulin secretion.^20^ Briefly, cholesterol accumulation alters lipid raft composition and membrane fluidity, resulting in reduced glucose transporter membrane levels, increased glucokinase (GCK) retention in insulin granules, and alteration of spatial organization of L-type voltage-gated Ca2+ channels (VGCC) and K+ ATP channels.^21^ Notably, a question arises whether atorvastatin-promoted LDL-C uptake can achieve the pathological effects of cholesterol accumulation on these cells. Our study finds that atorvastatin combined with LDL-C impairs GSIR at a 2 nM dose of glucose, suggesting that atorvastatin-promoted LDL-C uptake may have harmful effects. To address the underlying mechanism, further studies should use isolated pancreatic β-cells or other β-cells lines to elucidate the dose-response relationships between atorvastatin, LDL-C, and GSIR. Given that the LDLR pathway is also responsible for the internalization of modified LDL-C,^22^ circulating modified LDL-C can cause cytotoxicity in pancreatic β cells.

In addition to affecting lipid metabolism, some studies also report that statins can promote the β cells’ mass.^23,24^ In contrast, we found that low doses of atorvastatin have no significant effect on cell proliferation, but a 100 nM dose inhibits MIN6 cell proliferation. As previously described,^14^ a 100 nM dose of atorvastatin reduces 32.12% of cell viability and 34.07% of insulin secretion rate, suggesting that this dose of atorvastatin causes the cytotoxic effect on pancreatic β cells. In contrast, atorvastatin combined with a low dose of LDL-C can obviously promote the proliferation of MIN6 cells. Of note, it cannot be simply speculated that this effect is good for pancreatic β cells. Because atorvastatin combined with LDL-C may act as a low-dose of toxic agent, cells respond to the minor toxic effect by elevated viability and proliferation, and this phenomenon is therefore called hormesis.^25^ If the addition of LDL-C alters the effects of atorvastatin on pancreatic β cells, it is hard to believe that LDLR not be involved in this process. Our study reveals that atorvastatin regulates LDLR expression, increasing LDLR on the cell surface without altering total protein levels. Given the limitations of the current method, future research should consider isolating cell membranes to investigate LDLR protein expression more accurately. In light of total LDLR protein and mRNA expressions remaining unchanged, atorvastatin may regulate the numbers of LDLR on the cytomembrane by affecting protein post-translational modifications. In these pathways of LDLR post-translational modification, PCSK9 is the most important post-translation regulatory protein for LDLR, which binds LDLR and escorts them to lysosome and prevents its recycling to the cell surface^11^. When PCSK9 monoclonal antibody inhibitors are combined with statins, it has been suggested that it reduces LDL-C by 60% and cardiovascular events by 15–20%.^26^ β-cells constitute the cell type richest in PCSK9 expression, closely followed by hepatocytes, and a possible toxic effect is found in the PCSK9 knock-out mice and human pancreatic β-cells line via excessive cholesterol internalization in β-cells.^27,28^ More importantly, pancreatic PCSK9-null mice exhibit normal blood PCSK9 and cholesterol levels but are glucose intolerant and have defective insulin secretion, indicating that in situ PCSK9 plays a key role in maintaining the function of pancreatic β cells.^29^ Intriguingly, our study finds that PCSK9 protein expression in MIN6 cells is reduced by atorvastatin incubation, but such PCSK9 reduction is neutralized by the addition of LDL-C, indicating that statin-induced PCSK9 secretion is regulated by exogenous cholesterol. The observed effect may be due to the interplay between LDL-C uptake and atorvastatin’s action on LDLR, which in turn affects the synthesis and degradation of PCSK9. Notably, this phenomenon should be further investigated using isolated islets, and the underlying mechanism requires further elucidation. Unlike PCSK9, IDOL is not easily influenced by atorvastatin regardless of LDL-C levels. IDOL is also called Mylip and is another important regulator of the post-transcriptional control of LDLR abundance, which can bind LDLR to induce its degradation via E3 ubiquitylation^30^. The differential effects of atorvastatin on PCSK9 and IDOL expression further elucidate the regulatory mechanisms of LDLR on the cell surface, providing insights into how statins may modulate LDLR levels and function in pancreatic β-cells. 6, plasma PCSK9 and insulin levels in mice were also reduced by oral atorvastatin, suggesting that PCSK9 is a critical mediator in influencing cholesterol uptake and the functional dynamics of pancreatic β-cells. Indeed, a real-world study shows that people treated with PCSK9 inhibitors are associated with mild hyperglycemia,^31^ but there is no firm proof to support the association between PCSK9 inhibitors usage and the risk of NOD.^32^ However, based on our findings, the pathological effect of PCSK9 on pancreatic β-cells should be considered in the pathogenesis of statin-associated diabetes.

## Conclusion

This study provides novel insights into the effects of atorvastatin on cholesterol metabolism in pancreatic β-cells and its potential implications for statin-associated diabetes risk. Our findings show that atorvastatin increases the uptake of LDL-C in MIN6 cells by regulating LDLR levels on the cell surface, which is combined with a notable decrease in PCSK9 expression. This mechanism does not lead to cholesterol accumulation but may impair pancreatic islet function, particularly under conditions of added exogenous cholesterol. Our findings highlight the importance of PSCK9 in patients receiving statin therapy, especially those patients with a higher risk of diabetes. Further research is necessary to fully elucidate the mechanisms by which statins influence pancreatic β-cell function and to develop strategies to mitigate the risk of statin-associated diabetes.

### Limitations

This study provides valuable insights into the effects of atorvastatin on LDL receptor regulation and pancreatic β-cell function; however, several limitations should be acknowledged. First, the experiments were conducted using MIN6 cells, which, while widely used as a β-cell model, may not fully replicate the physiological responses of primary pancreatic islets. Future studies using isolated islets or in vivo models are needed to validate these findings in a more physiologically relevant setting. Second, while we observed atorvastatin-induced changes in LDL-C uptake and PCSK9 expression, the long-term effects of these alterations on β-cell function and glucose homeostasis require further investigation. Lastly, potential compensatory mechanisms, such as cholesterol efflux or metabolism, were not directly assessed and should be explored in future studies to provide a more comprehensive understanding of the impact of atorvastatin on pancreatic β-cells.

## Supplementary Material

Supplemental Material

Supplemental Material

## Data Availability

All data supporting the conclusions of this article are available from the corresponding author on reasonable request.

## Abbreviation

ACVD: atherosclerotic cardiovascular diseases
LDL-C: lowering low-density lipoprotein cholesterol
T2D: type 2 diabetes
LDLR: LDL receptors
PCSK9: proprotein convertase subtilisin/kexin type 9
GSIR: glucose-stimulated insulin release
NOD: new onset diabetes
TC: total cholesterol
FC: free cholesterol
CE: cholesterol ester
IDOL: inducible degrader of LDL receptor
HMGCR: HMG-CoA reductase
GCK: glucokinase
VGCC: voltage-gated Ca2+ channels.
