# Supplementary material for: Atorvastatin enhances LDL receptor-mediated LDL-C uptake and modulates PCSK9 protein expression in pancreatic β-cells
Source: Islets. 2025 Mar 16;17(1):2479906. doi: 10.1080/19382014.2025.2479906 (PMC11913382; doi:10.1080/19382014.2025.2479906)
Supplement: Supplemental Material [file KISL_A_2479906_SM5186.docx]

| Fig.1A | Normality |  |  |  |  |  | Homogeneity of variance |  |
| --- | --- | --- | --- | --- | --- | --- | --- | --- |
|  | TC |  |  |  |  |  | Brown-Forsythe test |  |
|  | Shapiro-Wilk test |  |  |  |  |  | F (DFn, DFd) | 0.7015 (4, 10) |
|  | W | 0.9595 | 0.7812 | 0.8895 | 0.7992 | 0.9153 | P value | 0.6085 |
|  | P value | 0.6129 | 0.0703 | 0.3529 | 0.1126 | 0.436 | P value summary | ns |
|  | Passed normality test (alpha=0.05)? | Yes | Yes | Yes | Yes | Yes | Are SDs significantly different (P < 0.05)? | No |
|  | P value summary | ns | ns | ns | ns | ns |  |  |
|  | CE |  |  |  |  |  |  |  |
|  | Shapiro-Wilk test |  |  |  |  |  | Brown-Forsythe test |  |
|  | W | 0.9868 | 0.7831 | 0.8892 | 0.7974 | 0.9179 | F (DFn, DFd) | 0.5831 (4, 10) |
|  | P value | 0.7804 | 0.0748 | 0.352 | 0.1083 | 0.4451 | P value | 0.6821 |
|  | Passed normality test (alpha=0.05)? | Yes | Yes | Yes | Yes | Yes | P value summary | ns |
|  | P value summary | ns | ns | ns | ns | ns | Are SDs significantly different (P < 0.05)? | No |
|  | FC |  |  |  |  |  |  |  |
|  | Shapiro-Wilk test |  |  |  |  |  | Brown-Forsythe test |  |
|  | W | 0.8675 | 0.8929 | 0.9271 | 0.8547 | 0.9894 | F (DFn, DFd) | 0.8064 (4, 10) |
|  | P value | 0.2886 | 0.3631 | 0.4778 | 0.253 | 0.8028 | P value | 0.5485 |
|  | Passed normality test (alpha=0.05)? | Yes | Yes | Yes | Yes | Yes | P value summary | ns |
|  | P value summary | ns | ns | ns | ns | ns | Are SDs significantly different (P < 0.05)? | No |
| Fig.1B | Normality |  |  |  |  |  | Homogeneity of variance |  |
|  | TC |  |  |  |  |  |  |  |
|  | Shapiro-Wilk test |  |  |  |  |  | Brown-Forsythe test |  |
|  | W | 0.9997 | 0.9277 | 0.9559 | 0.8156 | 0.7946 | F (DFn, DFd) | 1.151 (4, 10) |
|  | P value | 0.9657 | 0.4801 | 0.5958 | 0.1523 | 0.1016 | P value | 0.3878 |
|  | Passed normality test (alpha=0.05)? | Yes | Yes | Yes | Yes | Yes | P value summary | ns |
|  | P value summary | ns | ns | ns | ns | ns | Are SDs significantly different (P < 0.05)? | No |
|  | CE |  |  |  |  |  |  |  |
|  | Shapiro-Wilk test |  |  |  |  |  | Brown-Forsythe test |  |
|  | W | 0.9666 | 0.9335 | 0.949 | 0.9868 | 0.8602 | F (DFn, DFd) | 1.245 (4, 10) |
|  | P value | 0.6488 | 0.502 | 0.565 | 0.7802 | 0.2681 | P value | 0.3529 |
|  | Passed normality test (alpha=0.05)? | Yes | Yes | Yes | Yes | Yes | P value summary | ns |
|  | P value summary | ns | ns | ns | ns | ns | Are SDs significantly different (P < 0.05)? | No |
|  | FC |  |  |  |  |  |  |  |
|  | Shapiro-Wilk test |  |  |  |  |  | Brown-Forsythe test |  |
|  | W | 0.9067 | 0.9796 | 0.9643 | 0.9979 | 0.8811 | F (DFn, DFd) | 1.309 (4, 10) |
|  | P value | 0.4072 | 0.7262 | 0.6369 | 0.9118 | 0.3275 | P value | 0.3311 |
|  | Passed normality test (alpha=0.05)? | Yes | Yes | Yes | Yes | Yes | P value summary | ns |
|  | P value summary | ns | ns | ns | ns | ns | Are SDs significantly different (P < 0.05)? | No |

| Normality |  |  |  |  | Homogeneity of variance |  |
| --- | --- | --- | --- | --- | --- | --- |
| Fig.2A |  |  |  |  |  |  |
| Shapiro-Wilk test |  |  |  |  | Brown-Forsythe test |  |
| W | 0.8935 | 0.9353 | 0.8964 | 0.9283 | F (DFn, DFd) | 1.965 (3, 34) |
| P value | 0.2168 | 0.5024 | 0.2321 | 0.431 | P value | 0.1378 |
| Passed normality test (alpha=0.05)? | Yes | Yes | Yes | Yes | P value summary | ns |
| P value summary | ns | ns | ns | ns | Are SDs significantly different (P < 0.05)? | No |
| Fig.2B |  |  |  |  |  |  |
| Shapiro-Wilk test |  |  |  |  |  |  |
| W | 0.8097 | 0.893 | 0.8231 | 0.8351 | Brown-Forsythe test |  |
| P value | 0.0363 | 0.2497 | 0.0503 | 0.0671 | F (DFn, DFd) | 5.085 (3, 28) |
| Passed normality test (alpha=0.05)? | No | Yes | Yes | Yes | P value | 0.0762 |
| P value summary | ns | ns | ns | ns | P value summary | ns |
|  |  |  |  |  | Are SDs significantly different (P < 0.05)? | No |

| Normality |  | Homogeneity of variance |
| --- | --- | --- |
| Fig.3A |  |  |
| Shapiro-Wilk test |  |  |
| W | N too small | N too small |
| P value |  |  |
| Passed normality test (alpha=0.05)? | | |
| P value summary |  |  |
| Fig.3B |  |  |
| Shapiro-Wilk test |  |  |
| W | N too small | N too small |
| P value | |  |
| Passed normality test (alpha=0.05)? | |  |
| P value summary | |  |

| Normality |  |  |  |  | Homogeneity of variance |  |
| --- | --- | --- | --- | --- | --- | --- |
| Fig.4A |  |  |  |  |  |  |
| Shapiro-Wilk test |  |  |  |  | Brown-Forsythe test |  |
| W | 0.9872 | 0.9615 | 0.7903 | 0.995 | F (DFn, DFd) | 0.6411 (3, 8) |
| P value | 0.783 | 0.6226 | 0.0915 | 0.8648 | P value | 0.6097 |
| Passed normality test (alpha=0.05)? | Yes | Yes | Yes | Yes | P value summary | ns |
| P value summary | ns | ns | ns | ns | Are SDs significantly different (P < 0.05)? | No |
| Fig.4D |  |  |  |  |  |  |
| Shapiro-Wilk test |  |  |  |  | Brown-Forsythe test |  |
| W | 0.8421 | 0.9049 | 0.8929 | 0.7693 | F (DFn, DFd) | 0.1834 (3, 8) |
| P value | 0.2196 | 0.4014 | 0.3631 | 0.0431 | P value | 0.9047 |
| Passed normality test (alpha=0.05)? | Yes | Yes | Yes | Yes | P value summary | ns |
| P value summary | ns | ns | ns | ns | Are SDs significantly different (P < 0.05)? | No |

| Normality |  |  |  |  | Homogeneity of variance |  |
| --- | --- | --- | --- | --- | --- | --- |
| Fig.5A |  |  |  |  |  |  |
| Shapiro-Wilk test |  |  |  |  | Brown-Forsythe test |  |
| W | 0.9472 | 0.9287 | 0.7604 | 0.7675 | F (DFn, DFd) | 0.5453 (3, 8) |
| P value | 0.557 | 0.4838 | 0.123 | 0.0991 | P value | 0.665 |
| Passed normality test (alpha=0.05)? | Yes | Yes | Yes | Yes | P value summary | ns |
| P value summary | ns | ns | ns | ns | Are SDs significantly different (P < 0.05)? | No |
| Fig.5B |  |  |  |  |  |  |
| Shapiro-Wilk test |  |  |  |  | Brown-Forsythe test |  |
| W | 0.9382 | 0.7626 | 0.9963 | 0.94 | F (DFn, DFd) | 1.592 (3, 8) |
| P value | 0.5202 | 0.128 | 0.8833 | 0.5275 | P value | 0.266 |
| Passed normality test (alpha=0.05)? | Yes | Yes | Yes | Yes | P value summary | ns |
| P value summary | ns | ns | ns | ns | Are SDs significantly different (P < 0.05)? | No |
| Fig.5C |  |  |  |  |  |  |
| Shapiro-Wilk test |  |  |  |  | Brown-Forsythe test |  |
| W | 0.9989 | 0.7786 | 0.9592 | 0.8485 | F (DFn, DFd) | 0.2969 (3, 8) |
| P value | 0.9368 | 0.0644 | 0.6118 | 0.2364 | P value | 0.8268 |
| Passed normality test (alpha=0.05)? | Yes | Yes | Yes | Yes | P value summary | ns |
| P value summary | ns | ns | ns | ns | Are SDs significantly different (P < 0.05)? | No |
| Fig.5D |  |  |  |  |  |  |
| Shapiro-Wilk test |  |  |  |  | Brown-Forsythe test |  |
| W | 0.9725 | 0.999 | 0.9982 | 0.9421 | F (DFn, DFd) | 0.4622 (3, 8) |
| P value | 0.6819 | 0.9389 | 0.9196 | 0.5358 | P value | 0.7164 |
| Passed normality test (alpha=0.05)? | Yes | Yes | Yes | Yes | P value summary | ns |
| P value summary | ns | ns | ns | ns | Are SDs significantly different (P < 0.05)? | No |
