# Supplementary material for: Atorvastatin enhances LDL receptor-mediated LDL-C uptake and modulates PCSK9 protein expression in pancreatic β-cells
Source: Islets. 2025 Mar 16;17(1):2479906. doi: 10.1080/19382014.2025.2479906 (PMC11913382; doi:10.1080/19382014.2025.2479906)
Supplement: Supplemental Material [file KISL_A_2479906_SM5176.docx]

LDLR 140 kDa

GAPDH 36KDa

180

130

100

70

Repeat 1 Repeat 2 Repeat 3

180


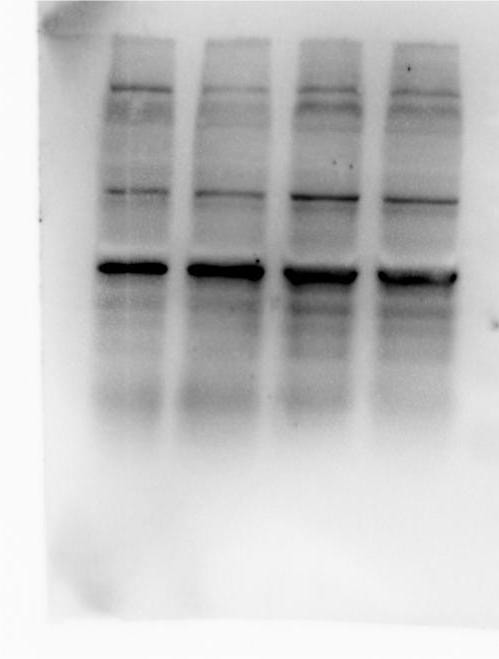

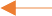

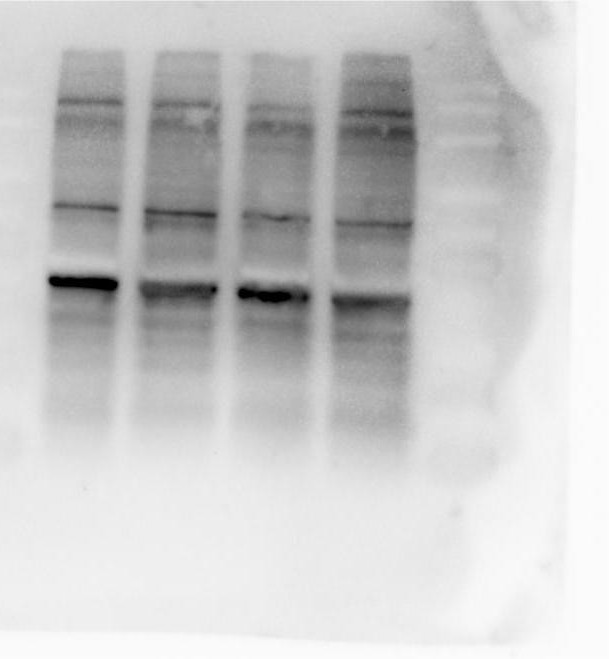

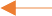

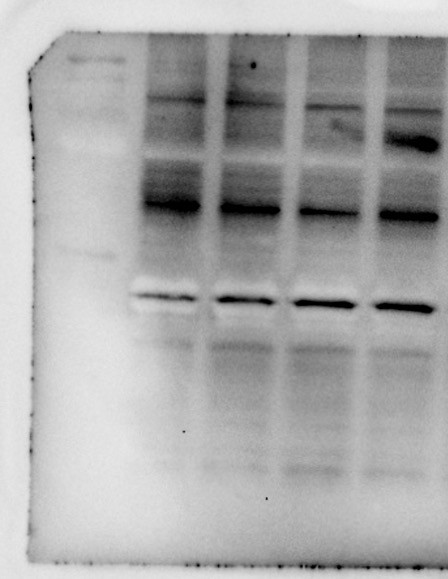

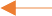


180

130

100

70

130

100

70


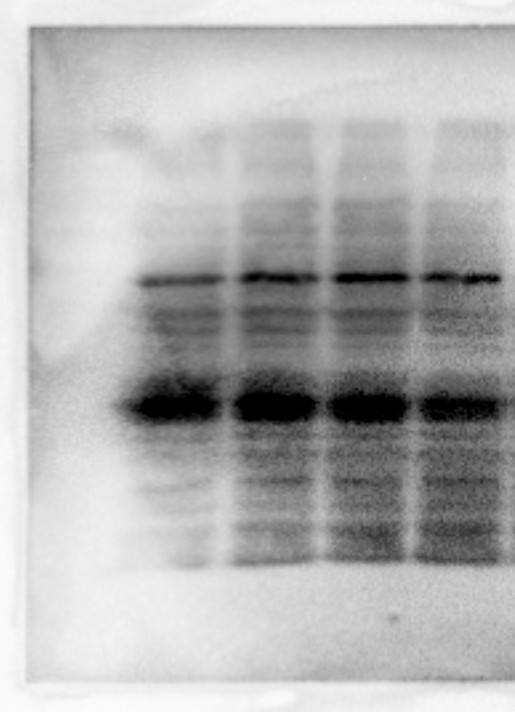
40


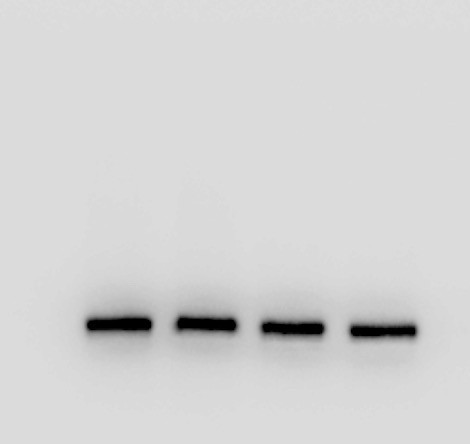


40

35


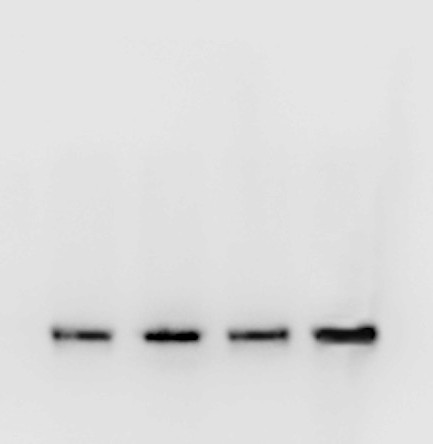


40

35

35

0, 1, 10, 100 nM atorvastatin

Repeat 1 Repeat 2 Repeat 3

LDLR 140 kDa

GAPDH 36KDa

180

130

100

70

180

130


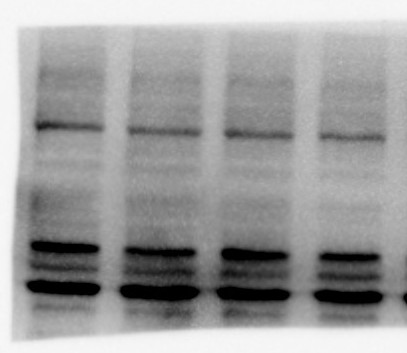

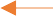

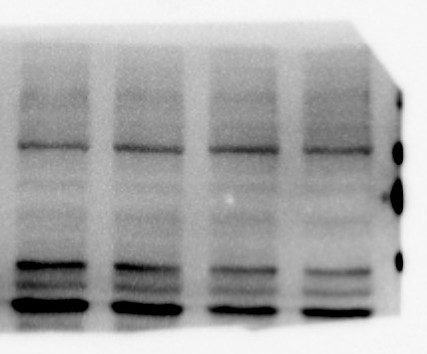

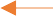

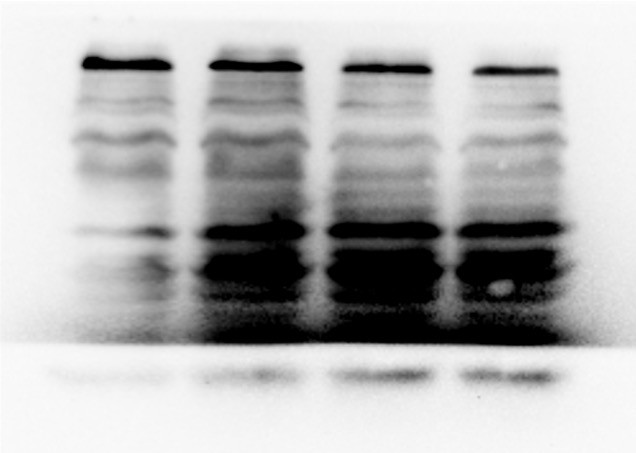

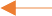


180

130

100

70

100


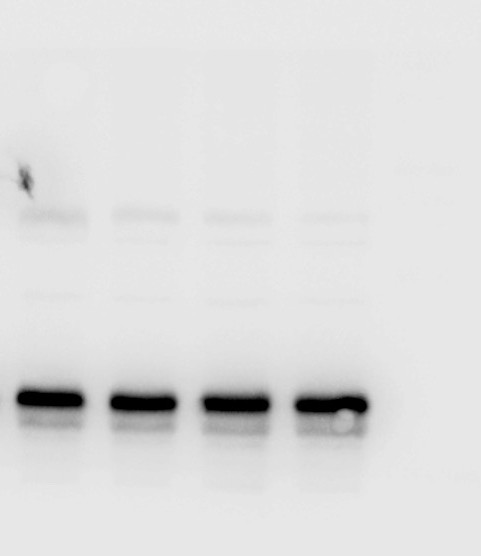
70


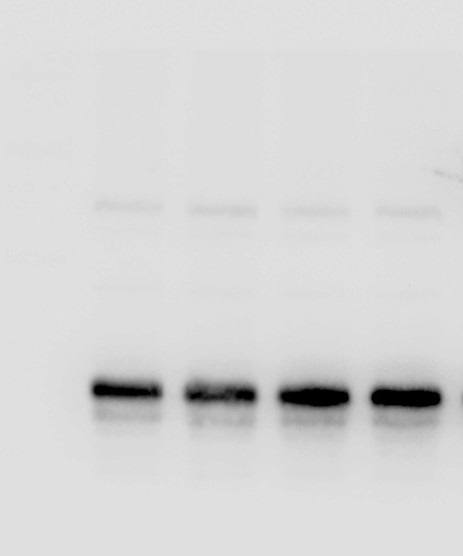


40

35


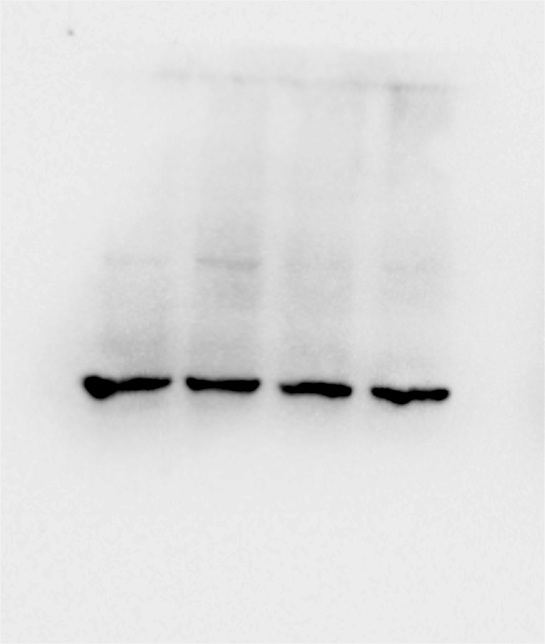
40 40

35 35

0, 1, 10, 100 nM atorvastatin with10 ug/mL LDL

PCSK9 70KDa

GAPDH 36KDa

180

130

100

70

Repeat 1

180

130

100

70

40

35

Repeat 2 Repeat 3


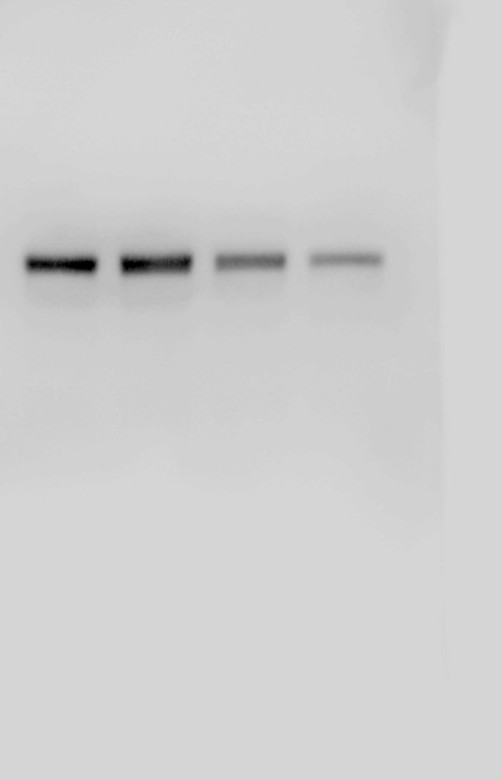
180


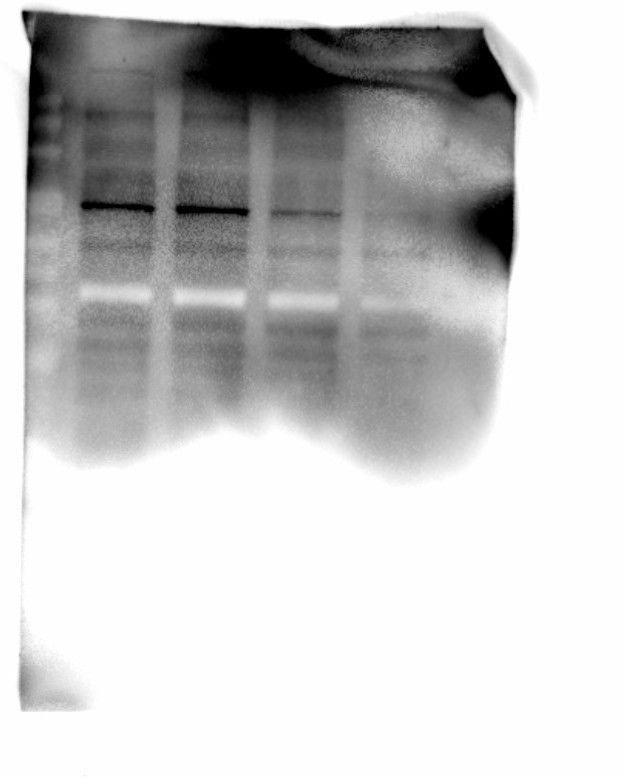

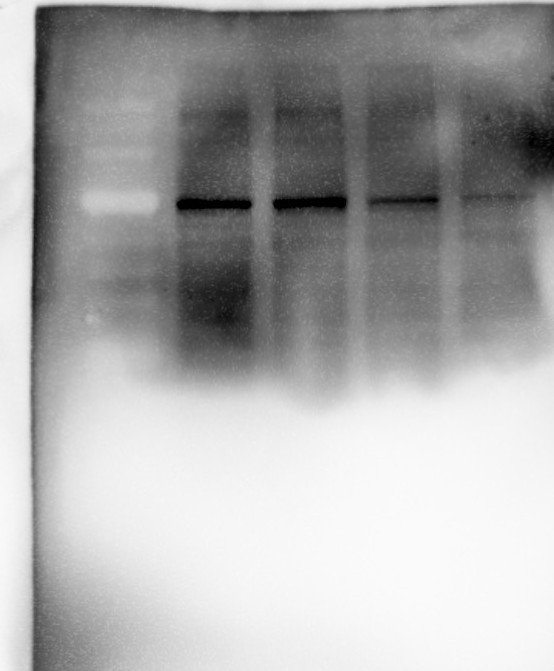


130

100

70

40

35


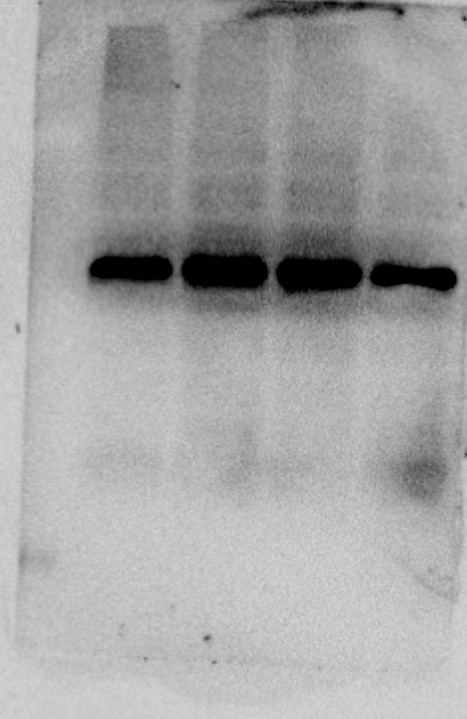

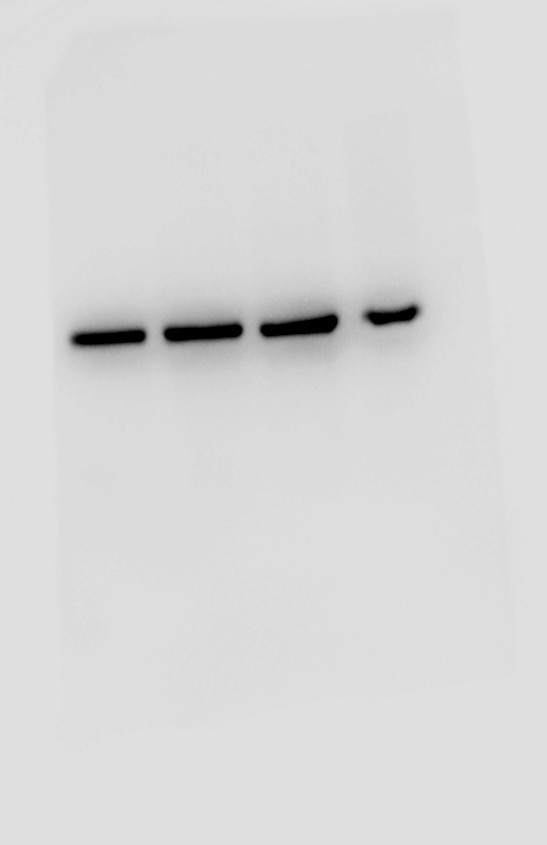
40

35

0, 1, 10, 100 nM atorvastatin


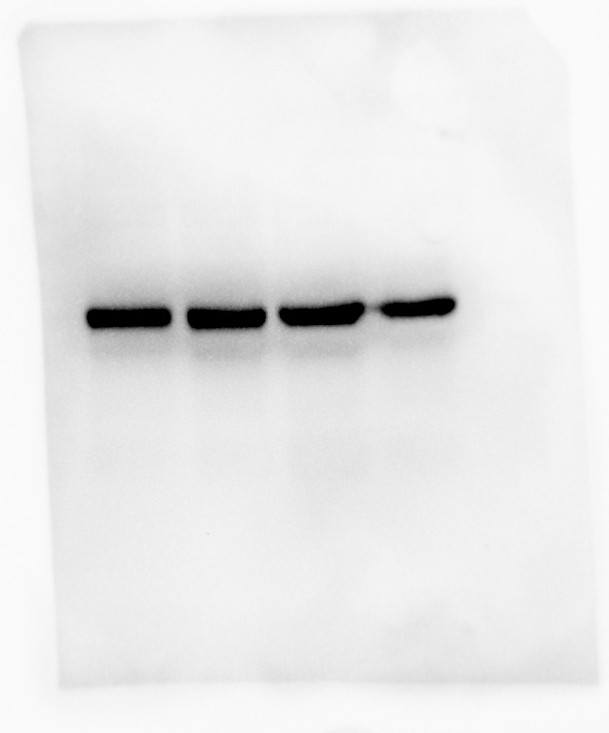


40

35

# Figure 5 PCSK9 original western blot for three repeats


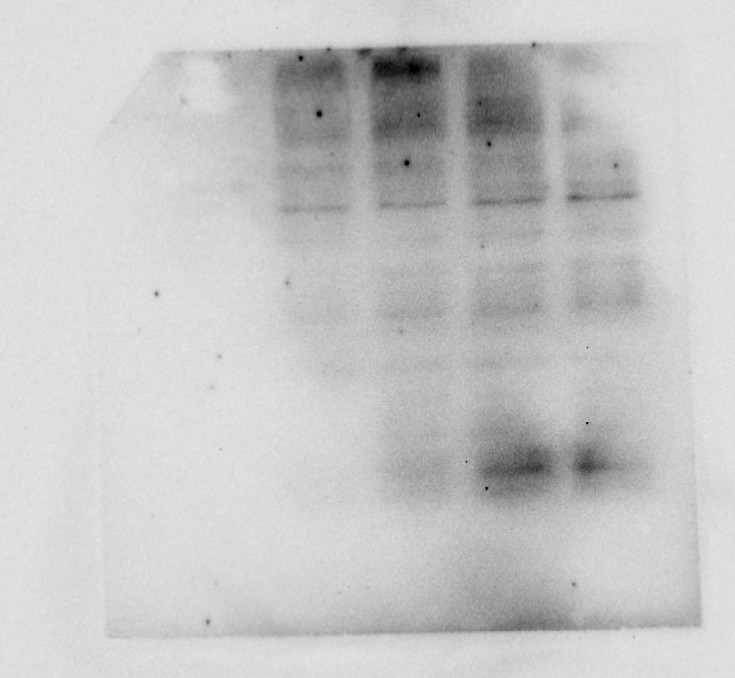

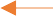


100

70

Repeat 1 Repeat 2 Repeat 3

Pcsk9 70KDa


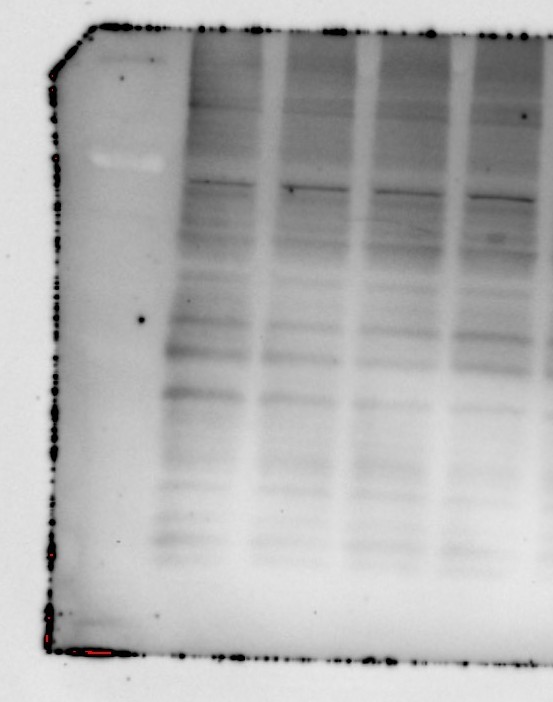

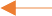


100

70

100

70


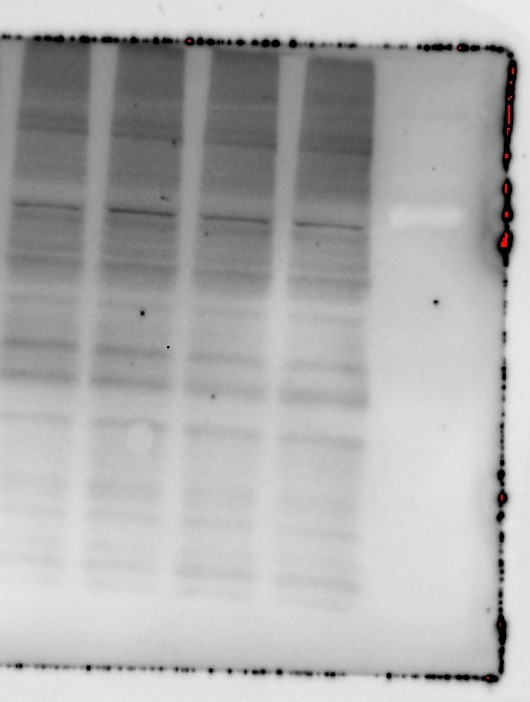

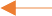


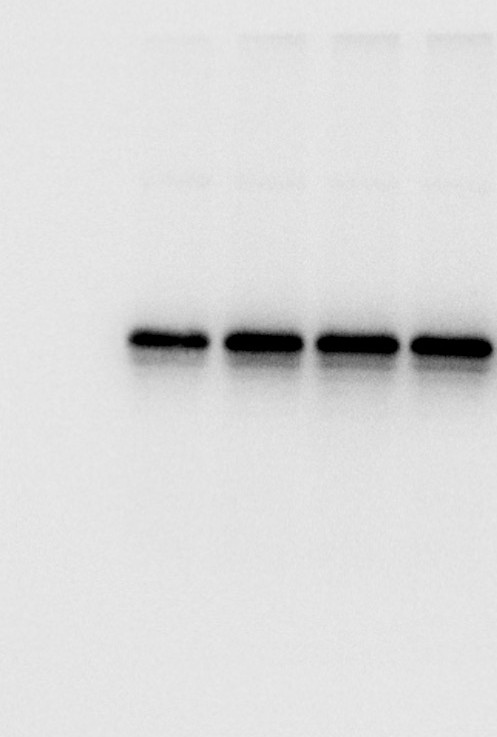

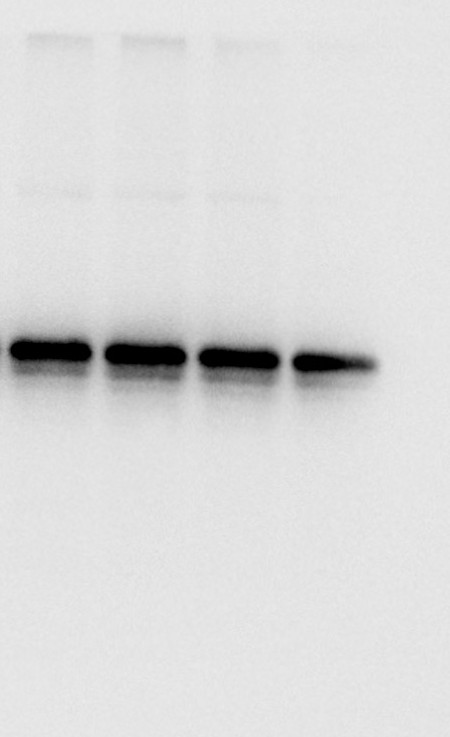

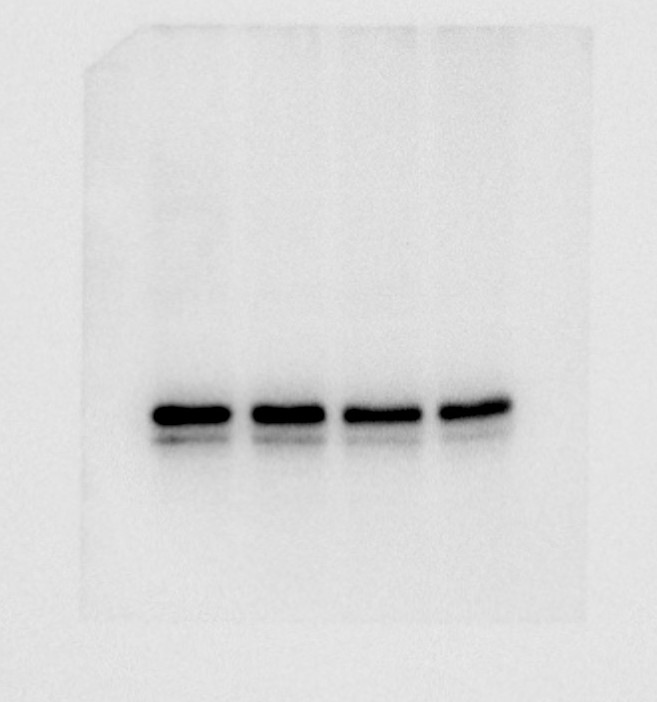
40 40 40

GAPDH 36KDa 35 35 35

0, 1, 10, 100 nM atorvastatin with10 ug/mL LDL

Repeat 1 Repeat 2 Repeat 3

IDOL 50KDa

100

70

50

100


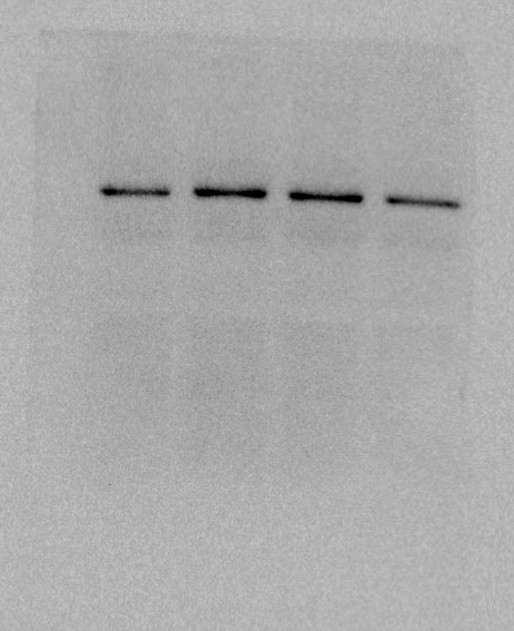

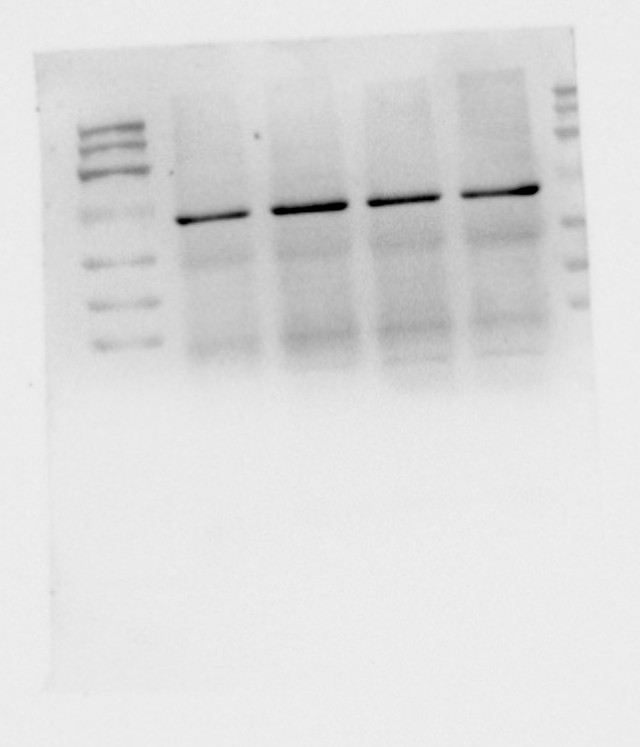
70

50

100


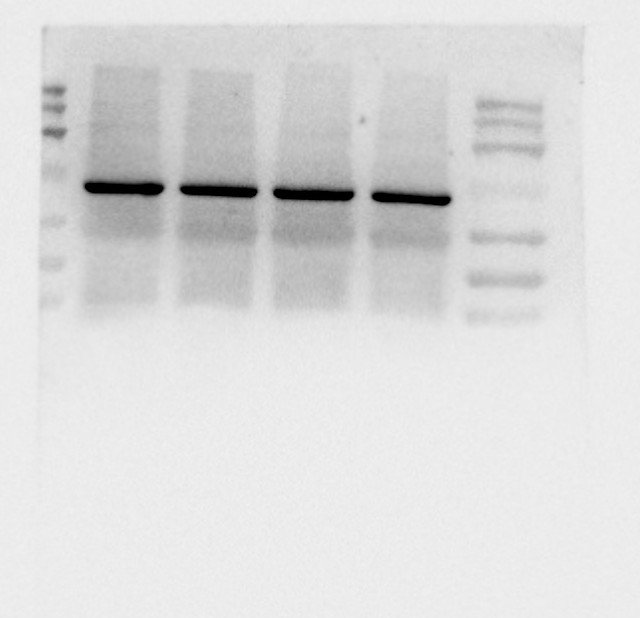
70

50


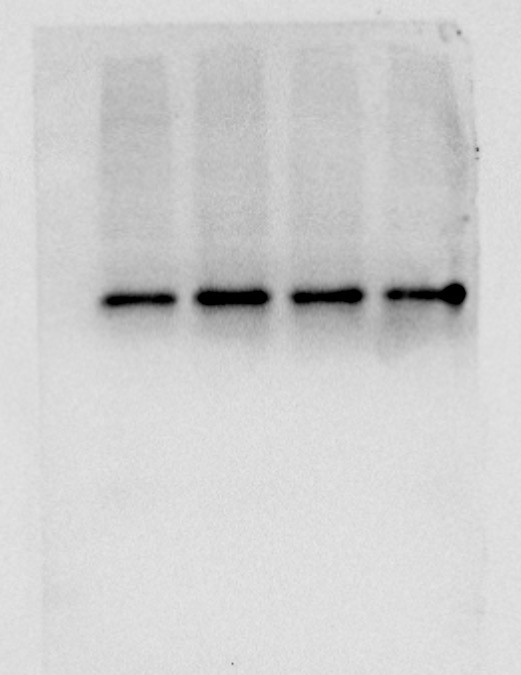

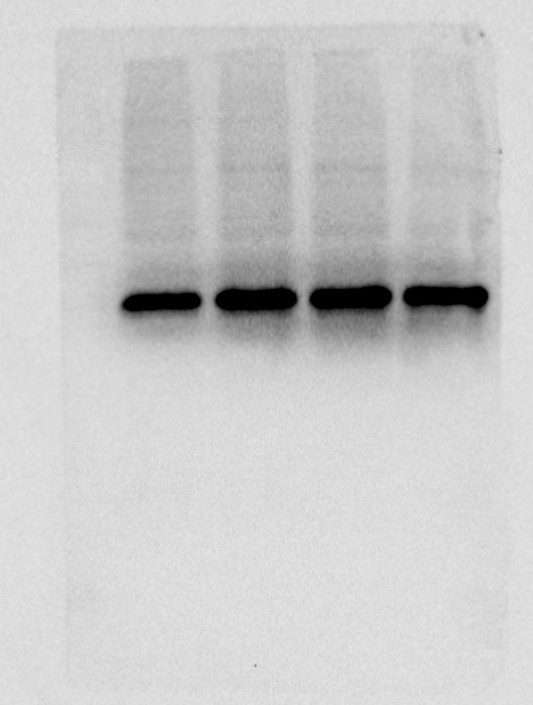

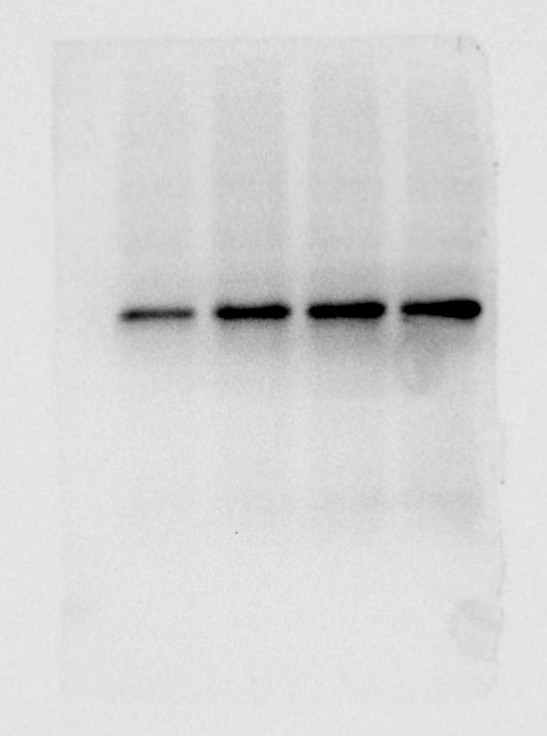
40 40 40

GAPDH 36KDa 35 35 35

0, 1, 10, 100 nM atorvastatin

Repeat 1 Repeat 2 Repeat 3

IDOL 50KDa

GAPDH 36KDa

100


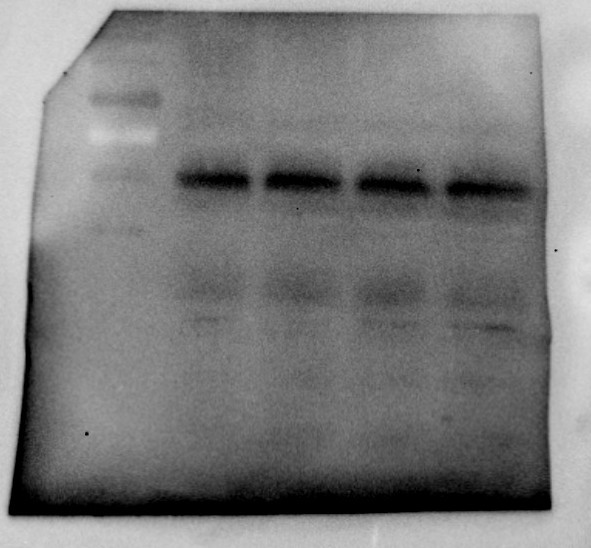
70

100

70

50

50

100


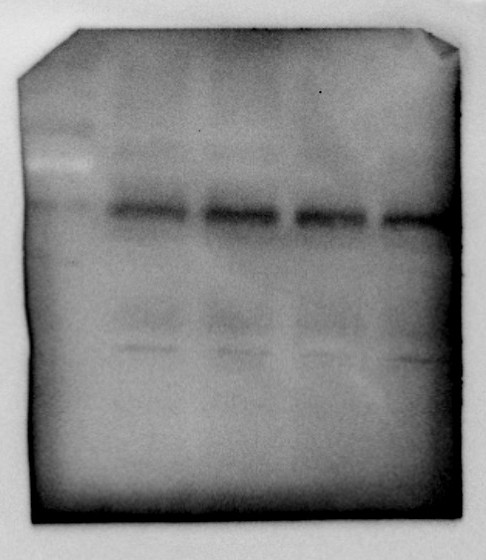
70

50


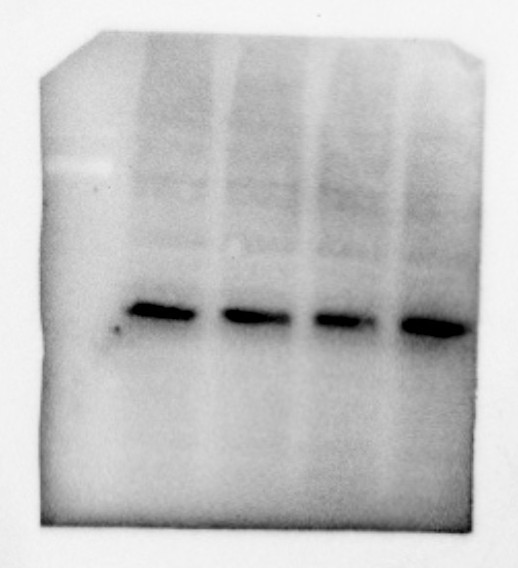
40

35

100

70


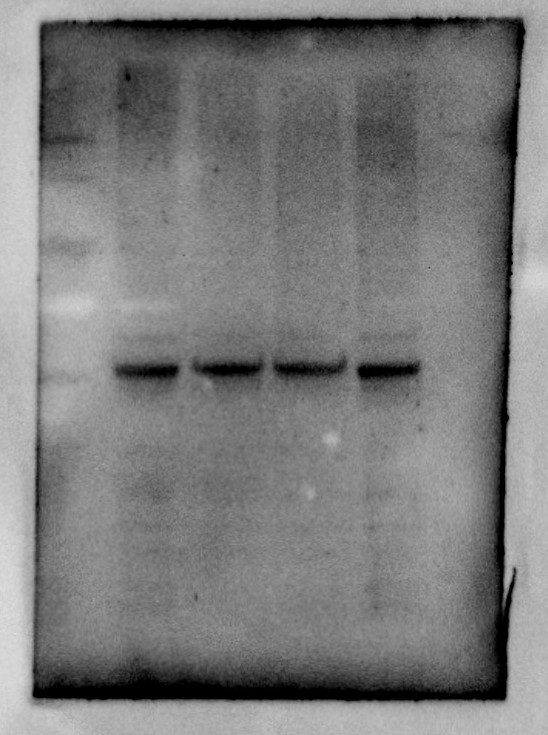

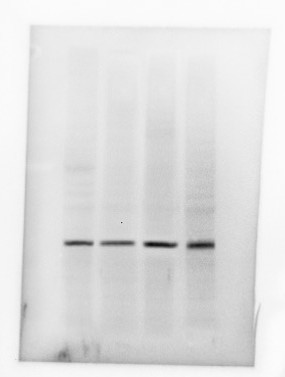


40

35

50

0, 1, 10, 100 nM atorvastatin with10 ug/mL LDL


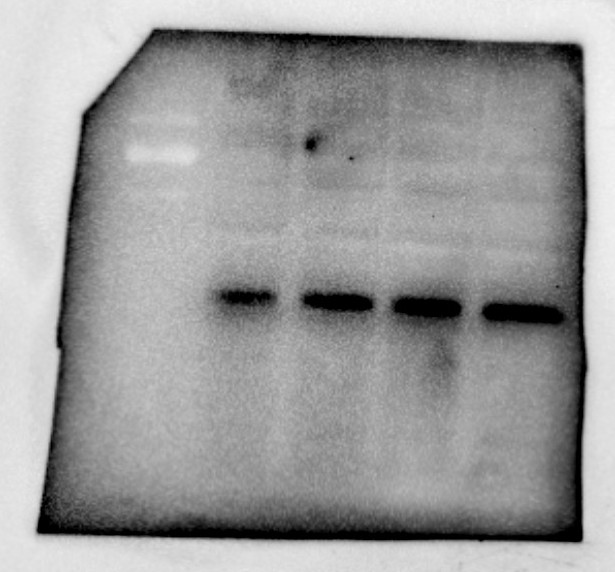


40

35
